# Adsorption of Bisphenol A from Water Using Chitosan-Based Gels

**DOI:** 10.3390/gels11030180

**Published:** 2025-03-05

**Authors:** Ludmila Aricov, Anca Ruxandra Leontieș

**Affiliations:** “Ilie Murgulescu” Institute of Physical Chemistry, Romanian Academy, Splaiul Independenţei 202, 060021 Bucharest, Romania; laricov@icf.ro

**Keywords:** chitosan, gel, pollutant, bisphenol A, absorption

## Abstract

The comonomer bisphenol A (BPA) finds applications in the plastics industry, where it is used in the production of polycarbonates, plastics, PVC, thermal paper, epoxy and vinyl ester resins, and polyurethane. The water, with which many of these materials come into contact, is one of the main sources of human exposure to BPA. When ingested or touched, BPA can damage organs, disrupt the endocrine and immune systems, generate inflammatory responses, and be involved in genotoxic processes. Therefore, the need to develop effective techniques for removing BPA from aqueous environments is imperative. This paper provides a comprehensive review regarding the effective removal of BPA from water, focusing on the performance and adsorption mechanisms of various adsorbents based on chitosan and chitosan composites. The chemical and physical factors, adsorption kinetics and models governing the adsorption process of BPA in chitosan materials are also examined. This review outlines that, despite considerable progress in the absorption of bisphenol using chitosan gels, further research is necessary to assess the efficacy of these adsorbents in treating real wastewater and in large-scale manufacture.

## 1. Introduction

Bisphenol A (BPA) and analogs belong to a class of chemical compounds widely used for consumer goods’ production [1,2]. BPA, the most prevalent bisphenol, has a molecular structure containing two phenol functional groups connected by carbon units resulting from the condensation of acetone with two phenol molecules. From its synthesis until today, numerous studies have depicted BPA as a widespread disruptor of terrestrial and aquatic ecosystems, adversely affecting human and animal health and compromising plants through hormonal imbalance and bioaccumulation [1,3]. BPA is challenging to eliminate due to its ubiquitous presence in water and its chemical characteristics that facilitate accumulation in living organisms. Consequently, effective treatment of water sources and removal strategies to reduce the related negative effects of BPA are particularly important.

Commonly used physical treatment processes for removing BPA from water and wastewater are adsorption, membrane filtration, and electrocoagulation. These methods are attractive because they are versatile, simple to use, and highly effective. However, there are disadvantages associated with the above methods that need to be considered. Membrane downsides may include reduced removal efficiency, a high risk of membrane fouling, and greater energy and maintenance expenses. Adsorption is currently the most commonly used strategy. Among the various water treatment methods, flocculation–coagulation is the simplest and cheapest method. BPA is extracted from the contaminated sludge during the first treatment stage using physicochemical techniques (flocculation or coagulation). Rather than occurring during the secondary treatment stage, this process occurs in the primary stage where the documented removal rate is only 1%, indicating that coagulation has limited effectiveness in removing BPA. During the secondary treatment phase, BPA undergoes several complex processes, including oxidation, enhanced oxidation, membrane technologies, and adsorption; taken together, they show amazing removal efficiency. Among the various water treatment methods, flocculation–coagulation is the simplest and cheapest method [4].

Adsorption emerges as a particularly promising environmentally friendly method for the efficient elimination of BPA from aqueous environments [5]. Its versatility, wide application, affordability, and practicality establish it as a preferred and efficient method for pollutant removal. Numerous substances ranging from natural materials (such as clays, straw, feathers and coffee grounds [6,7,8]) to natural polymers (such as chitosan, chitin, alginate, cellulose, hemicellulose, starch, etc. [9]) and activated carbon [6], polyacrylic acid and synthetic polyvinyl (polyacrylic compounds, vinyl acid, polyvinyl alcohol, epoxy resin, polyaniline, etc.) or combinations thereof [10,11,12] are known as adsorbents of BPA from aqueous media. The properties of chitosan, such as safety, compatibility, cost effectiveness, adaptability, sustainability, and simplicity of modification, make it a promising material in a variety of applications for water purification [13,14]. These features not only emphasize its potential but also validate its inclusion in scientific and industrial research, making it a topic of interest in this study. However, chitosan has several drawbacks, including low chemical and mechanical strength, which complicates the separation of absorbed ions. Fortunately, chemical and physical changes may address these issues [13,15]. For instance, chitosan-based magnetic compounds are efficient adsorbents that show excellent separation and reusability properties for BPA [16,17]. Adding biomaterials from food and agricultural waste, like orange peel, coffee grounds, bamboo, bone, olive pomace, and more, to chitosan is another way to make biocomposites that are better at removing harmful pollutants from water [18]. Other researchers improved chitosan’s absorption properties by adding natural or synthetic compounds, including poly(N-isopropylacrylamide) [19], phytic acid [20,21,22], alginate [23], gelatin [24], acrylic acid [25], poly(methacrylate) [26], polyvinyl alcohol [27], etc.

Consequently, in this review, we will examine the efficacy of chitosan and chitosan composites as adsorbent agents for the removal of BPA from water. This review is confined to the data derived from gel-based chitosan materials; thus, the discussion that follows will focus exclusively on these materials and their adsorption characteristics. The primary aims are to define recent developments related to the BPA adsorption applications of these polymeric materials and to provide pertinent insights regarding their most significant characteristics. The influence of diverse parameters, including the properties of chitosan, the conditions for modification, the variables of the process, and the experimental settings employed in batch systems, on biosorption is discussed and reviewed. This paper meticulously describes the equilibrium and kinetic models, alongside the adsorption mechanism stated for sorption onto chitosan, which are crucial for determining the adsorption capacity and for the design of water treatment processes.

## 2. BPA Risk Assessment

**Figure 1:**
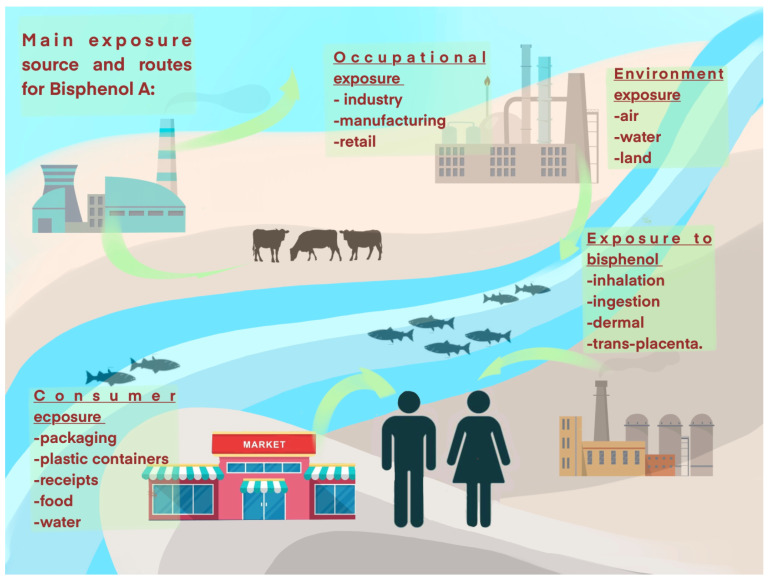
The main ways through which humans, animals, and plants come into contact with bisphenols.

From its synthesis over 120 years ago until today, BPA production has come to a total of 6.4 million tons worldwide and is estimated to reach 9.3 million tons in 2030 [28]. The first report of BPA toxicity appeared in the early 1930s, when a group of scientists found that BPA may be considered a synthetic estrogen [29]. Starting in the 1940s, new types of plastics appeared on the market, namely, polycarbonates and epoxy resins based on BPA. Even though BPA toxicity was signaled and the leaching out of plastic was known, the plastics were used for consumer goods’ production without any safety requirements [30]. The 70 years following BPA’s introduction as a prime substance for plastics came with controversy and debates regarding its toxic effects. Starting in 2009, after a long and difficult process between consumers, industries and FDA, regulations regarding children’s exposure to BPA emerged [31]. Afterwards, Canada, the EU and China proposed strict regulation regarding BPA use in products destined for human [32,33,34]. As more scientific evidence about BPA’s harmful effects grew, the EU set the daily limit at 0.2 ng per kilogram of body weight per day in 2020. This is 20,000 times lower than the daily limit of 4 μg per kilogram of body weight per day in 2015 [35]. Even though it is controlled, BPA is still found in building and packaging materials, electronics, medical equipment, thermal paper, and even vehicle components. People can be exposed to bisphenols by breathing them in, touching them, or eating them. Therefore, Figure 1 shows the main ways that humans, animals and plants are exposed to bisphenols.

**Figure 2:**
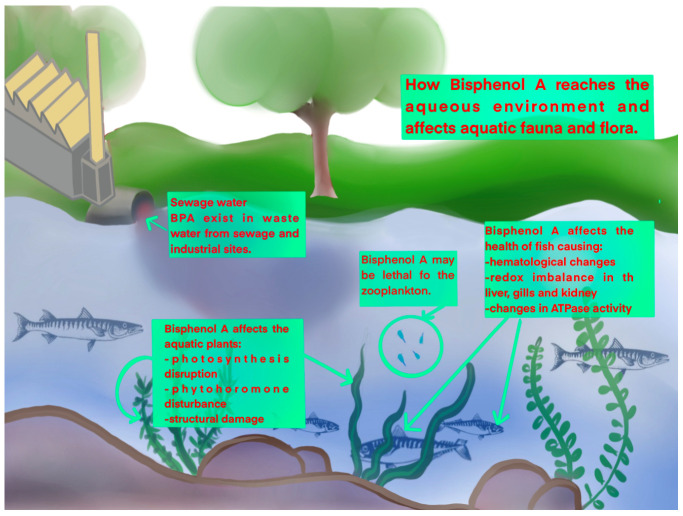
The effects of BPA toxicity on aquatic life.

Due to BPA’s widespread use, the environment (soil, rivers, and lakes) and oceans are contaminated. The main pathways to environmental pollution with BPA are water discharge from municipal treatment plants and landfill leaching [36]. Significant concentrations of BPA in leachate have been detected in landfills from southeastern Europe, with estimated values between 0.70 and 2.72 mg/L [37]. Furthermore, Yamamoto et al. found high concentrations of BPA in landfill leachate in Japan, ranging from 0.0013 to 17.20 mg/L [38]. Goeury et al. investigated the steroid hormones and BPA levels in surface waters and suspended sediments of Quebec, Canada [39]. The study found that BPA was one of the most common pollutants in dissolved phase samples, with levels as high as 62 ng/L. In sediments, the levels ranged from 2.4 ng/g to 228 ng/g depending on where the samples came from. The authors concluded that BPA concentration from samples was affected by dilution, particularly in the summer months where lower concentrations were detected as compared with the spring or winter months. Moreover, water samples from the lower part of the Danube River Middle Basin (either surface water or wastewater) had an average BPA concentration of 16 ng/L to 224 ng/L [40]. Other studies on the European environment reported a concentration of 39 μg/L of BPA in the lowland aquifers of Berkshire, UK [41]. For groundwater samples, in Poland, a concentration of 6.88 μg/L was reported, while in Portugal, a maximum concentration of BPA in river water of 0.3066 µg/L has been found [42]. Surface waters from Japan, Korea, China, and India contained significant amounts of BPA [43]. The Chennai rivers in India had the highest concentrations of BPA, ranging from 54 to 1950 ng/L, followed by Korea at 4.6 to 272 ng/L, Japan at 6.5 to 120 ng/L, and China at 43 to 73 ng/L. Sea water samples collected along the Black Sea coast of Romania showed that the mean amount of BPA is around 165 ng/L. According to research on BPA levels in the Black Sea, Bosphorus, and Marmara Sea, data showed that the levels reach 14.76 × 10^3^ ng/L [44]. Furthermore, the result of water contamination with BPA is identified in plants, animals, and even humans. According to EU recommendations, the range of BPA in aquatic plants, typically from 1 to 100 mg/L, is considered to have a harmful impact on the aquatic environment [45]. Figure 2 illustrates the impact of BPA toxicity on aquatic life.

**Figure 3:**
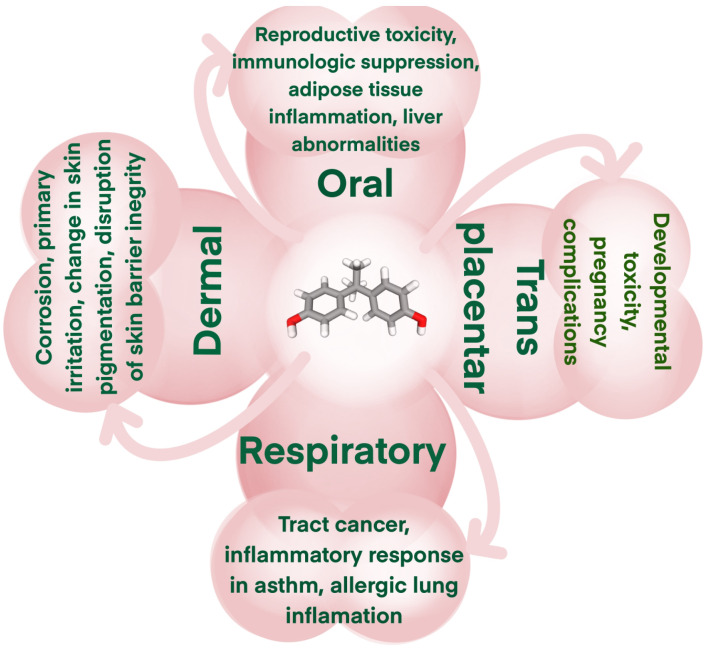
The illustration of the relationship between BPA and human diseases.

BPA affects not only plants but also river and marine animals. For example, this harmful compound was detected at up to 396 ng/g in fish and shellfish from the Pearl River Estuary in China and as high as 755.7 ng/g in Polish fish meat [46,47]. Numerous studies reported that BPA affects fish and other marine animals by interfering with their metabolism, cortisol levels, axonal growth, musculature, and motor behavior [48,49,50,51]. Various biomonitoring investigations in humans have demonstrated that substantial BPA use has resulted in widespread exposure and associated health hazards [52]. The Human Biomonitoring for Europe initiative assessed BPA and other endocrine disruptors in 9493 participants from 21 countries [53]. A staggering 92% of the population exhibited urinary BPA levels exceeding 11.5 ng/L, a value above the permissible limit by more than 57 times. The study’s results are significant because they allow for linking BPA levels in humans to information about their diet and health. This makes it possible to link internal doses to possible exposure sources and health outcomes. These effects can be seen from the time of pregnancy, when BPA exposure is linked to a higher risk of bronchial asthma in children. Moreover, this problem extends beyond humans, as BPA harms offspring in several animal species from early developmental stages [54]. Other consequences of animal exposure to BPA include a reduction in offspring numbers, abnormal larval development, and delayed emergence of insects. At the cellular level, BPA binds to estrogen receptors in the same way as estradiol and diethylstilbestrol and generates cellular responses. Figure 3 illustrates how BPA can lead to diseases in humans, including thyroid and reproductive dysfunction, diabetes, and obesity. BPA also impacts the cardiovascular system.

Furthermore, BPA might change DNA methylation patterns, histones, and RNA expression, which would lead to epigenetic changes and possibly help cancer grow in people [55].

Consequently, eliminating BPA from water is crucial for environmental protection, and efficient water treatment and filtering techniques, with adsorption being fundamental for ensuring human health.

## 3. Chitosan Adsorbents in Water and Wastewater Treatment

Chitosan is considered an optimal water treatment agent due to its chemical structure, biodegradable nature, and low cost. In a conventional water treatment plant, several primary operations, including coagulation, precipitation, flocculation, and sedimentation, can be performed with chitosan. Research data show that chitosan’s ability to coagulate and flocculate may be a key step for treating wastewater [56]. Interestingly, this natural polymer changes its properties depending on the pH of the medium in which it is solubilized. This means that it dissolves in acid and has a cationic character, which lets it interact with anionic compounds in ways that are wanted in processes like adsorption, coagulation, and flocculation [57]. Chitosan can be combined with metal cations or organic compounds like anionic dyes when the pH is neutral because its amino groups are not protonated. Adsorption happens when amino groups become positively charged. This lets the molecules interact with metallic and organic anions. Following this step, the anions neutralize the amino groups, causing the coagulation phenomenon. Under fast stirring, the surface charges of colloidal suspensions (often found in wastewaters) are limited; therefore, they allow for aggregation. The aggregation of neutralized particles results in an increased density, thereby causing sedimentation. The second stage is the flocculation of aggregated materials under slow stirring. Chitosan is a powerful flocculation agent because it has a high molecular mass, can form networks, and has a high degree of deacetylation. Table 1 shows some examples of how chitosan and modified chitosan can be used to effectively separate and clump together different types of wastewater substances, including oils, drugs, dyes, and more.

**Figure 4:**
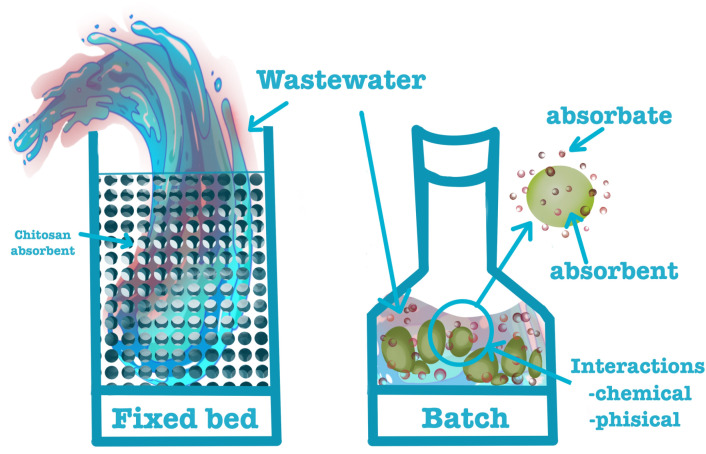
Illustration of the fixed bed and batch adsorption procedure.

Coagulation–filtration, although highly effective and rapid from a kinetic standpoint, cannot address all types of undesirable constituents in wastewater. An example is that of bisphenols. Nevertheless, we can employ adsorption to mitigate these undesirable chemicals. Current practice often uses the adsorption process for water and wastewater treatment. This process is energy-efficient, cost-effective, and versatile. Its flexible design and ease of operation make it possible to treat effluents that are odorless, colorless, and free of sludge [68,69]. When the attractive forces between molecules in the fluid phase and the solid surface are stronger than the attractive forces between molecules in the fluid phase, absorption may be physical. Consequently, a balance is reached between the molecules of the fluid that adhere to the solid adsorbent and the rest of the fluid phase. The attraction is mainly caused by Vander Waals forces. When chemical adsorption occurs, it requires the formation of new chemical bonds between the fluid (adsorbate) and the solid (adsorbent). In many circumstances, adsorption, whether physical or chemical, is an irreversible process, making it difficult to extract the adsorbate from the adsorbent. More often, for water purification, adsorption is used in fixed beds and in batch systems, as exemplified in Figure 4.

Continuous fixed-bed systems retain pollutants by passing the contaminated water across an adsorbent bed. A recent study demonstrated that a cross-linked chitosan/zeolite composite can be used as a fixed bed in a column for the removal of BPA from water [70]. The authors determined that with a hydraulic retention period of 0.8 hours, a pH = 5.1, and a BPA concentration of 1.636 mg/L, about 1.456 mg/L (89.0%) of BPA, had been removed by absorption. The Langmuir isotherm explains the removal of BPA micropollutants by chitosan/zeolite with an R^2^ of 0.973 and Q_max_ of 1.6. According to regeneration and desorption data, this type of fixed-bed column filled with cross-linked chitosan/zeolite could be used seven times without significantly modifying the removal efficiency.

More often, batch systems are used to examine how polluted water can be treated using an adsorbent material. The unique features and current research discoveries offer chitosan-based gels potential for treating wastewater in batch systems. Chitosan-based hydrogel materials are recognized for their ease of separation and handling, as well as their regeneration and recycling capabilities, making them suitable for extensive applications in water decontamination.

## 4. Main Parameters Affecting Modified Chitosan as Adsorbing Agent

Researchers frequently employ batch methods to examine the remediation of contaminated water using an adsorbent. Chitosan-based gels may be applied for wastewater treatment in batch systems due to their distinctive features and recent research discoveries [71]. To enhance the absorption rates of chitosan-based molecules, one must carefully study specific parameters in these systems. Among the main parameters studied, the following stand out for their significant influence on the removal of compounds by adsorption. The main parameters studied include pH and temperature, the duration of contact with the adsorbate, the concentration of both the adsorbent and the adsorbate, and perhaps most importantly, the type of polymer modification [72].

### 4.1. pH, Temperature, and Contact Time

The pH, temperature, and contact time directly influence the adsorption process of chitosan-based materials. Changes in solution pH, which have an effect on both the relative charges of the adsorbent and adsorbate molecules, have the potential to influence the interaction and adsorption capacity of the molecules involved. At an acidic pH, chitosan is predominantly protonated, resulting in a positively charged surface that enhances the adsorption of negatively charged contaminants. In alkaline conditions, chitosan typically undergoes deprotonation, resulting in a negatively charged surface that facilitates the adsorption of positively charged pollutants [73]. Meanwhile, elevated temperatures may enhance the rate of chemical reactions and the diffusion of pollutants, leading to improved adsorption. Conversely, under specific conditions, higher temperatures can facilitate or hinder the adsorption process [74]. Contact time is the length of contact time between the adsorbent and the solution and strongly influences the adsorption kinetics. According to research data, longer contact times may provide better adsorption equilibrium, while shorter contact times may make it less effective at removing pollutants [75,76,77]. In order to clarify the adsorption process, it is important to examine the following thermodynamic characteristics: free energy (ΔG), enthalpy change (ΔH), and entropy (ΔS). Negative enthalpy values imply physical adsorption, whereas positive values suggest chemical adsorption. Furthermore, changes in free energy show that the process occurs spontaneously. Examining these aspects is crucial for enhancing adsorption and understanding key process factors such as temperature, interactions, and spontaneity [69].

Chitosan and its derivatives have been the focus of numerous investigations addressing their potential for pollutant removal, particularly with regard to dyes, heavy metals, and pharmaceuticals. These investigations have addressed the influence that pH, temperature, and contact time have on chitosan and its derivatives [73,78,79,80,81]. A review published in 2021 indicated that for chitosan–graphene oxide materials, the optimal adsorption capacity of heavy metal ions occurs at pH = 5–7 [80]. Similarly, at a pH of 5, the silica gel–chitosan combination was able to reach its maximum adsorption capacity for both Cu^2^⁺ (870 mg/g) and Pb^2^⁺ (330 mg/g) [82], while in the case of chitosan–silica gel, the adsorption decreases for cadmium and nickel ions as the temperature increases [83].

The pH and temperature significantly influenced the adsorption of various dyes, including reactive blue, red, yellow, and black, when utilizing simple or modified chitosan [73]. The findings indicated that lower pH values are associated with increased adsorption capacities, while the optimum temperature was between 20 and 60 °C. A remarkable maximum adsorption capacity of 1100 mg/g for reactive black 5, at pH = 3, was achieved using flaked chitosan [84]. The adsorption efficacy was enhanced by increasing the pH within the range of 2.8 to 5.6 during tetracycline adsorption utilizing chitosan in the presence of Cu(II) [81]. The literature indicates that materials derived from chitosan demonstrate optimal effectiveness in adsorbing pollutants at pH values ranging from 3 to 7 and temperature values between 25 and 60 °C.

### 4.2. Chemical Composition

Another major factor that can be modified to improve the effectiveness of absorbents is their chemical composition. This can be accomplished by adding functional groups and modifying the surface of the material. Chitosan-based hydrogel polymers find extensive application in the field of water decontamination due to their accessibility, ease of separation, manageable handling, and capacity for regeneration and recycling [85]. On the other hand, chitosan-based gels have certain drawbacks as well. They have low mechanical strength and do not remain stable in extreme environmental conditions, such as high pH and temperature [86]. In order to overcome these challenges, there are a number of various strategies that may be used to improve the physicochemical features of hydrogels. These approaches include functionalization with nanomaterials and magnetic particles, reinforcement with fibers, treatment with cross-linking agents, and mixing with other polymers that have the opposite charge [13,15,86].

Chitosan-based gels can be modified through chemical or physical alterations of the polymer chains to enhance the previously mentioned properties. These commonly used chemical methods are quaternization, amidation, and oxidation. Adding quaternary ammonium, amine groups, and carbonyl groups to the chitosan structure is one of the methods. This improves the structure–activity relationships. By doing so, one may obtain materials with enhanced biocompatibility and antimicrobial activity [87,88], pH sensitivity [89], and improved mechanical strength [90].

Chitosan-based gels may be combined with various materials, from quantum to microscale [91], natural or synthetic [69], to create composite gels with the desired characteristics [91,92]. Common preparation procedures of chitosan gels encompass physical mixing, chemical cross-linking and the sol-gel procedure [93]. A large number of literature reviews describe, in depth, the possible modifications of chitosan so that gels may be obtained and underline their numerous applications in various technologies. Radhakrishnan and Panicker provided an updated review concerning the basic structure, functional modifications, characterization, processing, and uses of chitosan materials. Furthermore, the recent initiatives aimed at augmenting the performance of chitosan and the underlying mechanisms responsible for the enhancements in its features were examined. Their study delineated the future paths for novel and sustainable chitosan-based biomaterials [94]. In another review, Gonçalves et al. highlighted the significant properties of chitosan that enable diverse modification, hence broadening its uses. Chitosan’s efficacy in adsorbing pollutants, especially in complex water treatment methods, was emphasized. Their study highlighted chitosan-based hybrid materials, such as nanocomposites, hydrogels, membranes, films, sponges, nanoparticles, microspheres, and flakes, as novel substitutes for conventional chemical-based adsorbents [69]. Yazdi et al. examined the various routes for sintering of chitosan-based composites and the influence of several factors, including pH, temperature, adsorbent dose, adsorption mechanisms, and adsorbent renewal. The potential and technological barriers for enhancing the performance of chitosan-based composites in practical applications at a pilot or industrial scale were also examined [95]. Keshvardoostchokami et al. investigated the removal of nitrogen-containing pollutants by chitosan and chitosan derivatives. The authors reviewed numerous physically and chemically modified chitosan-based adsorbents (chitosan beads, cross-linked chitosan, chitosan–polymer composites, chitosan–inorganic material composites, and chitosan–metal complexes) and provided a thorough analysis of the current state of the art while also highlighting the deficiencies in knowledge concerning the classification, preparation, characterization, and essential properties of these materials. In a comprehensive analysis, the authors examined the possible use of chitosan gels in water treatment, namely, for the removal of impurities such as dyes, heavy metals, and different inorganic and organic substances [91].

## 5. BPA Removal by Adsorption Processes in Chitosan and Chitosan-Based Gels

**Figure 5:**
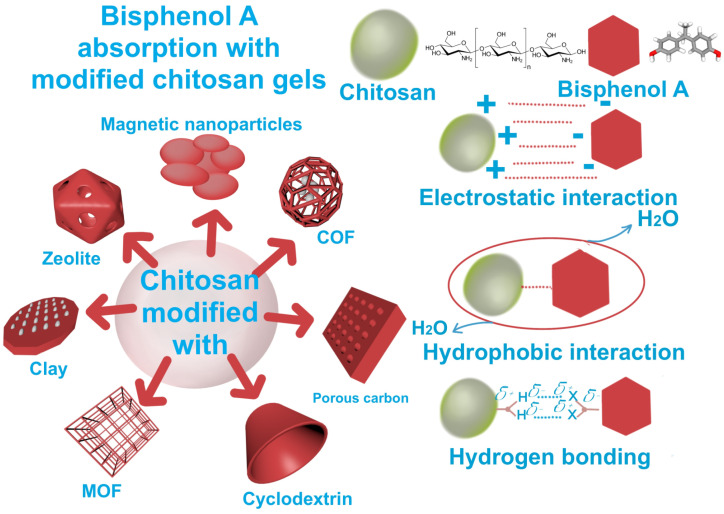
Diagrams based on potential processes for the adsorptive elimination of BPA using various adsorbents.

The enhancement of chitosan’s absorption capacity has emerged as an important field of investigation within the research community [9]. This can be accomplished through the modification of chitosan using a variety of natural or synthetic chemical agents [69]. Specifically, concerning the absorption of BPA, gel-based chitosan materials like cyclodextrin, metal–organic frameworks, and magnetic particles exhibit positive results. Frequently, the primary factors shaping the absorption mechanism of BPA in chitosan gel may arise from electrostatic interactions (π–π interactions), hydrogen bonds (acid–base), and hydrophobic interactions (such as pore filling). Figure 5 illustrates various materials commonly employed to enhance the absorbent characteristics of chitosan in the extraction of BPA from water, along with the primary interactions occurring between the chitosan gel surface and BPA molecules.

The effectiveness of several chitosan solutions, including those derived from commercially available chitosan and those generated from waste seafood shells, in removing BPA from water was thoroughly examined by Dehghani et al. [72]. Ideally, the chitosan concentration should be 0.06 g/L in order to obtain the 0.1 mg/L BPA maximum removal rate adsorption at pH = 5 with a contact period of 75 min and an adsorbent dosage of 0.06 g/L. The authors observed a positive relationship between the removal effectiveness of the pollutant and the increase in the initial absorbent concentration and contact time. The maximum BPA adsorption capacity for the synthesized chitosan was 34.48 mg/g, whereas for commercial chitosan, it was 27.02 mg/g. The kinetics of both materials follow a pseudo-second order model, while the Langmuir model was used to describe the equilibrium absorption isotherm.

As far as BPA is concerned, Zhou et al. designed an adsorbent that has interpenetrating networks composed of poly(N-isopropylacrylamide) and chitosan [19]. Under optimal conditions (pH = 7–9 and 35 °C), the authors state that the absorption kinetics of BPA follow a kinetic pseudo-second-order model. At 25 °C, the sigmoidal-shaped isotherm for BPA adsorption was fitted by the Slips, while at higher temperatures, both the Slips and Freundlich models applied well. The authors concluded that this type of absorption force increases with the adsorbate concentration or ambient temperature and is governed by hydrophobic interactions and hydrogen bonding. Interestingly, in the practical application, the anion coexistence had no negative influence on the adsorption of BPA. Moreover, the hydrogel was able to maintain its adsorption–desorption ability up to 94% in five cycles when the regeneration agent was methanol.

Dissolving chitosan combined with bentonite in an acidic solution, followed by sodium hydroxide precipitation, produces a physical gel suitable for BPA adsorption from wastewater. Ahari et al. assessed the process and found that BPA adsorption in chitosan–bentonite gel is influenced by contact time, initial pollutant concentration, water pH, and temperature [96]. The composite showed a removal efficiency of 465.21 mg/g for BPA under optimal conditions with rapid absorption kinetics. The process was defined by the pseudo-second-order equilibrium model, indicating that chemisorption significantly contributes to absorption, a response influenced by electrostatic attractions, hydrogen bonding, and van der Waals forces.

When advanced oxidation processes are required for pollutants like BPA, we recommend a combination of techniques like degradation and absorption. To this end, a research study proposed a new material consisting of chitosan mixed with alginate for Fe (II) fixation. The material was obtained by a simple cross-linking reaction to activate persulfate [97]. The composite removed 75% of 0.44 mM BPA by degradation, while the rest accounted for adsorption. This phenomenon was ascribed to BPA interaction with hydroxyl and amine groups of chitosan.

Affinity and selectivity are advantageous characteristics when considering gels for BPA absorption. A promising method to obtain these characteristics involves using a water-compatible molecularly imprinted polymer derived from β-cyclodextrin modified with magnetic chitosan for the selective adsorption of BPA from water [98]. According to the study findings, the amino and hydroxy groups in the cyclodextrin cavity form water-compatible imprinting cavities. This makes the absorption and selective qualities better. The adsorption process of BPA is ascribed to hydrophobic interactions and hydrogen bonding, supported by electrostatic interactions and dipole–dipole interactions with the polymer. The adsorption kinetics followed a pseudo-second-order model, while the Langmuir isotherm model effectively described the equilibrium adsorption, with a maximum adsorption capacity of 105.5 mg/g for BPA at room temperature for the modified polymer.

Mixing hydrophobic and hydrophilic polymers, such as chitosan and polyvinyl alcohol, may produce absorbent materials with better properties. In this regard, a research group developed a unique bifunctional adsorbent that can remove BPA from aqueous media [99]. The scientists included activated carbon into the polymer mixture in order to enhance its mechanical properties. The hydrogel that was generated was turned into beads, which reached a maximum adsorption capacity of 64.6 mg/g for BPA. The process of pollutant absorption into polymeric beads was spontaneous and endothermic, and it was characterized by the pseudo-second-order model. In this context, the Langmuir isotherm model performed better than the Freundlich model. Even when ions and humic acid were present, the high adsorption ability towards BPA (50 mg/g) was still maintained. Furthermore, the hydrogel absorbent was stable for five cycles in a row, demonstrating that it can be reused multiple times.

A group of researchers assessed the ability of chitosan versus vanillin-modified chitosan to absorb emerging contaminants, including BPA [100]. The results indicated that BPA absorption aligns with a pseudo-second-order model, with an adsorption velocity constant for BPA of 1.08 × 1016 g/(mg min), while a linear isotherm characterizes the experimental adsorption data on biosorbents. The research emphasized that chitosan modified with vanillin efficiently eliminates BPA and represents a viable alternative due to its ease of production and minimal reagent requirements.

Ali et al. introduced a biological sorbent composite containing chitosan, gelatin, and chlorella vulgaris that was impregnated with zinc oxide nanoparticles for the purpose of removing BPA from water [24]. The novel sorbent effectively removed 90% of 40.0 mg/L BPA at a pH = 4 and a contact time of 40.0 min. The study indicates that the adsorption of BPA in soft gel beads conforms to pseudo-second-order kinetics, with the adsorption isotherm represented by the Langmuir model. The authors concluded that the synthesized soft gel beads exhibited superior qualities, including an increased surface area, high adsorption capacity, reusability, and cost effectiveness.

Another study reviewed the ability of a composite consisting of chitosan covered with iron particles, graphene oxide, and clay in removing ethinylestradiol and BPA [101]. The investigators observed that the adsorption was caused by physisorption and was related to the creation of π–π interactions between the contaminants and the composite material. The selectivity experiments showed that the best results were achieved when the two pollutants had identical concentrations. In this case, almost 75% of the total amount of 0.010 mmol/L was eliminated.

A novel compound that combines a magnetic core with a chitosan shell that has been treated with β-cyclodextrin and cross-linked with graphene oxide was evaluated for its ability to adsorb BPA and BPF from wastewater [17]. They tested the ability of each adsorbent component to remove BPA and BPF from water under ideal conditions (adsorbent amount 20 mg/50 mL, adsorption time 60 min, temperature 30 °C, initial pollutant concentration 20 mg/L at pH = 7). Unfortunately, chitosan managed to absorb just 25% of the contaminants. At the same time, the synthesized material showed enhanced absorption rates in contrast to the raw materials, which most likely had limited removal capacities due to physical adsorption. The removal capability of the grafted material with β-CD was around 10–15% more than that of the unmodified material. Possibly the variations are caused by the hydrophobic cavity of β-CD, which is capable of producing stable complexes with bisphenol molecules. The experimental data are best matched by the pseudo-second-order kinetic model, which suggests that the studied material has many adsorption sites on its surface, with chemical adsorption being the dominant process and physical adsorption serving as a supplementary one. The highest BPF adsorption capacities for the composite material were determined to be 328.3 mg/g and 326.8 mg/g, respectively, using the Langmuir isotherm model. In addition, the authors demonstrated its usefulness in wastewater treatment using recycling testing, which demonstrated that the graphed material maintained over 80% efficacy in eliminating the bisphenols in five successive tests.

High adsorption, good water stability, easy separation, and excellent recyclability are the properties that researchers aim for. A composite material revealing these characteristics was synthesized by a team of researchers through a mixture of chitosan and alginate with aluminum-based metal–organic frameworks [102]. The pseudo-first-order model fit the absorption kinetics data the best. The bisphenol adsorption mechanisms on chitosan composite beads are mostly due to π–π stacking, hydrogen bonding, and cation–π interactions. The surface examination of the beads revealed that numerous pores were present. The pore presence was correlated with a large surface area that offers numerous adsorption sites for pollutant materials. In the composite beads, the hydrogen bonds formed by the nitrogen atoms of the polysaccharide and the hydroxyl group of BPA may be the removal of BPA. The ideal conditions found for the absorption were 50 mg/L of BPA with an aqueous pH = 7. The authors observed that absorption occurs rapidly (60 min) and reaches equilibrium in 18 h. From the standpoint of economic viability, the regeneration ability of adsorbents is a critical factor in water treatment. This outstanding material lost only 2.4% during the first adsorption/desorption cycle and, afterwards, remained constant until the fifth cycle, thus demonstrating the great stability and exceptional reusability of BPA.

Furthermore, in search of identifying economically viable and environmentally friendly materials, researchers are exploring alternative sources, encompassing renewable materials that exhibit both sustainability and cost effectiveness. The advancement of composite materials originating from waste biomass, including agricultural residues, represents a noteworthy field of inquiry. These materials can be meticulously engineered to exhibit unique properties that make them suitable for a diverse range of applications, including water remediation. Tan et al. contributed with a composite designed for this objective [103]. The authors put forth a proposal regarding the utilization of composite beads made from chitosan, bone, and bamboo biochar for the simultaneous removal of a metallic ion and bisphenol from aqueous solutions. The absorption studies revealed that the kinetics for BPA complied with a second-order pattern, along with the experimental data aligned with the Langmuir model. The substance exhibited a maximum absorption capacity of 140.30 mg/g, with the monomolecular layer adsorption mechanism prevailing as the primary chemisorption process. Moreover, subsequent to the adsorption process, the researchers employed the composite beads to degrade BPA in the presence of hydrogen peroxide, achieving an impressive removal rate of 99.2%. This effectively addresses the challenge of storing and disposing of used absorbents.

Kimura et al. contributed to a dual process utilizing chitosan and polyphenol oxidase for the absorption and elimination of bisphenol derivatives from aqueous environments [104]. The enzyme utilized catalyzes the oxidation of bisphenols into quinoline derivatives, and the incorporation of chitosan beads enhances the removal of reaction byproducts. Under ideal conditions (pH = 7–8, 30–40 °C), a total of seven bisphenols, including BPA, were entirely eliminated via the adsorption of quinone derivatives produced enzymatically on chitosan beads. Through a comparative analysis of chitosan beads and chitosan powder, the authors determined that the quinone oxidation process is expedited in the presence of chitosan beads; however, this enhancement is accompanied by enzyme inactivation, resulting in an incomplete removal of BPA. In instances where chitosan powder is employed, it was observed that complete removal occurred; however, an extended duration was required. The application of chitosan beads or chitosan powder for quinone adsorption in heterogeneous systems presents a more effective approach compared to the aggregation observed in homogeneous systems utilizing chitosan solutions for the removal of bisphenol oxidation byproducts.

BPA adsorption can also be further improved by refining membrane properties by developing new chitosan-based materials that exhibit a superior structural morphology, are environmentally sustainable, and, importantly, are economically viable while demonstrating high filtration capacity. Consequently, Zhao et al. engineered a remarkably effective separation membrane utilizing cellulose acetate and chitosan fibrous composites that have been treated with activated carbon [105] The recently developed membrane demonstrated remarkable efficiency, operating at a low feeding pressure of 0.1 mPa, achieving a high permeation flux of 9.09 × 10^3^ L/(m^2^ h^1^), and showing a significant rejection rate for BPA at 99.6%. The equilibrium of absorption was attained within a span of 3 min, with an adsorption capacity measured at 79.58 mg/g. The kinetics of absorption followed a pseudo-second-order model, and the experimental data conformed to the Langmuir isotherm model.

The modification of chitosan through polymer grafting has the potential to produce highly effective and adaptable absorbents for the purpose of water purification. The modification of chitosan via grafting can be accomplished through a range of techniques, including free radical polymerization, ring-opening polymerization, *γ*-radiation, or cationic polymerization, utilizing both “grafting from” and “grafting to” methodologies [106,107]. To date, there exist only a limited number of reports detailing the modifications of chitosan through the application of reversible addition–fragmentation chain transfer (RAFT). A recent study detailed the preparation of a biohybrid material through the modification of chitosan via RAFT polymerization in dispersed media, which was subsequently employed for the removal of BPA from water [106]. The chitosan beads, modified with synthetic poly(4-vinylpyridine), reached adsorption equilibrium in a BPA solution after 24 h. The concentration of beads at 10 mg/mL effectively eliminated up to 70% of a 100 ppm BPA solution. The authors observed that the adsorption capacity of their material was preserved at 61–62% of the initial adsorption across seven cycles of reusability. The sorption of BPA by the modified chitosan spheres can be attributed to the hydrogen bonding interactions occurring between the pyridine ring of poly(4-vinylpyridine) and the phenolic groups present in bisphenol. The results demonstrate that the RAFT technique outlined in this study is proficient in markedly enhancing the sorption properties of chitosan with respect to BPA.

In addition, the efficacy of adsorption in chitosan-based materials can be optimized by improving the mass transfer of analytes from the sample solution to the magnetic sorbents. A review of the literature reveals that the design and preparation of magnetic chitosan-based sorbents predominantly focuses on the absorption of toxic metal ions or dyes, while limited research on chitosan-based gels aimed at the removal of bisphenols are found. Hu et al. have adeptly created a composite material that integrates magnetic silica and chitosan, modified with n-dodecanal, to facilitate the absorption of bisphenol from aqueous solutions [108]. The adsorption rate reached 92% within a span of 15 min for a concentration of 10 mg/mL of BPA. The pseudo-second-order kinetic model proved suitable for BPA absorption, while the Freundlich isotherm model indicated the presence of multilayer adsorption behavior on the heterogeneous surface of the granular adsorbent. In summary, the data confirm that the magnetic composites serve as promising and effective agents for the removal of trace organic contaminants, including bisphenols, from aqueous environments.

A limited number of studies have examined the removal of bisphenol from aquatic systems using chitosan-based gels; hence, a significant body of research employing adsorption using this effective material remains necessary. Table 2 offers an examination of various studies employing a range of adsorbents, differing pH levels, and unique adsorption conditions, in conjunction with the kinetic and isothermal mechanisms that most precisely align with the experimental results relating to BPA removal via chitosan and chitosan-derived adsorbents.

For an effective chitosan-based gel adsorbent for BPA removal, researchers must consider the pH of the solution, which substantially affects the adsorption capacity of the adsorbent and the characteristics of the adsorbate. The BPA molecule has three primary forms dependent upon the solution’s pH. At a low pH (pH < 7), BPA predominantly exists in its neutral molecular form (BPA_0_), mostly unionized, hence its low solubility in water. Around pH ≈ 7, BPA starts to ionize, with the neutral form still predominating. Therefore, the ionization process begins to affect BPA’s solubility and interactions with other substances in water. At pH levels higher than 7, BPA exists primarily in its deprotonated form [BPA^-^], the maximum [BPA^-^] being attained at pKa1 = 9.59. Above this point, in alkaline environments, BPA ionizes more completely, increasing its solubility in water and its interaction with other charged species, BPA^2-^ appearing to be the majoritarian species at pKa2 = 10.2 [116]. When adsorption studies are involved, one must take into account the pH at the point of zero charge (pHzpc) of an adsorbent [27,111,112]. This parameter represents the pH at which the net surface charge of the adsorbent equals zero. Several adsorbent features depend on this parameter: adsorption mechanism (below the pHzpc, adsorbent surfaces are positively charged, attracting negatively charged pollutants, while over the pHzpc, the negatively charged will attract positively charged pollutants), optimal pH for adsorption (at or near the pHzpc adsorption capacity of the adsorbent for specific contaminants is maximum), surface charge characterization (helpful for designing effective treatment processes), and electrostatic interactions (important role in the adsorption process, especially for ionic species).

Simsek et al. conducted the removal of BPA using carbon-fiber-embedded chitosan and PVA composites, noting that the removal efficiency is greater at acidic pH and diminishes at basic pH [27]. Within the pH range of 3–9, the chitosan PVA material is unable to adsorb phenolic species via amino groups, the pHzpc being 8.51. The remaining material, the carbon fiber, is the only responsible for adsorption in this interval. Deprotonated BPA species repulsion and adsorbent functional group dissociation reduce adsorption system contact at pH levels of 9.0 and 11.0. The authors also state that the composite material surface becomes negatively charged above pH = 6.8. However, at pH = 9.0, electrostatic repulsion occurs as OH^−^ ions compete with bisphenolate anions for adsorption sites, thereby reducing adsorption effectiveness. The authors conclude that the composite materials containing both cationic and anionic functional groups are suitable for treating wastewater containing contaminants with differing charge characteristics.

Dehghani et al. showed that the highest adsorption of BPA onto chitosan-immobilized nanoscale zero-valent iron nanoparticles occurred at pH = 3 and decreased by rising pH levels [111]. Furthermore, they attributed the reduction in BPA adsorption to electrostatic repulsion and competition between hydroxide ions and BPA oxyanions for active adsorption sites. Their material had a pHzpc of 3, indicating that at this threshold, the surface of embedded chitosan is mostly characterized by positive charges at pH levels over 3, the surface is mostly characterized by negative charges. Consequently, the negative charge density on the adsorbent surface escalates with rising pH beyond pHzpc, leading to a reduction in BPA absorption.

Mohammadi et al. found a connection between pH and surface charge for magnetic multi-wall carbon nanotubes that had been treated with chitosan [112]. They also looked at how the nanotubes affected the rate at which BPA was absorbed. In summary, they observed the highest efficiency at pH = 5, and as the pH increased to alkaline, the removal efficiency decreased. We attributed this effect to the anionic configuration of BPA and the pHzpc of the material. Moreover, the research demonstrated that the pHzpc of carbon nanotubes is 8.5.

In conclusion, pHzpc helps optimize adsorption processes and analyze contaminant-adsorbent interactions. It helps choose the right conditions for pollutant removal and boosts the treatment system’s performance.

## 6. The Sustainability of Chitosan-Based Gel Adsorbents

Chitosan is a deacetylated form of chitin that is obtained from shrimp, crabs, and lobsters exoskeletons. The exoskeletons serve as byproducts, making chitin both readily available and sustainable. Recent studies have assessed the economic and environmental performance of converting shrimp wastes into valuable products. For example, Gómez-Ríos et al. compared the process technologies for chitosan production from shrimp shell waste and offered a complete techno-economic approach [117]. Their analysis of profitability, production costs, net present value, internal rate of return, and payback period concluded that chitosan production is profitable and cost-competitive within the geographic regional context, and they estimated that the mean gross margin may be 70%, while the mean internal rate of return is 25.5%. Cogollo-Herrera et al. also performed a techno-economic sensitivity analysis of the large-scale production of chitosan from shrimp shell wastes and identified the critical techno-economic variables as raw material costs, product selling price, and normalized variable operating costs, and recommended a chitosan cost of 50,000–120,000 USD/t [118].

Based on the idea that chitosan can be used instead of synthetic materials and that it completely breaks down in nature, it is clear that one of the best things about chitosan-based gels is that they can help with solving long-term problems, particularly those related to the remediation of water or environmental conditions. Also, solvents that are good for the environment, like water and acetic acid (which comes from plants and breaks down naturally), are the best choices for dissolving chitosan. Usually, chitosan-based gels are made by dissolving it in acidic media and then treating it with a coagulation solution or cross-linking agents [13,15]. For instance, biocompatible crosslinking agents can be used to make chitosan-based gels in a way that is good for the environment. Genipin is a chemical crosslinking agent that has many benefits, including being safe for long-term use and producing a stable product that can be used for cationic dye adsorption [119]. Interestingly, other authors showed that, depending on chitosan processing, the start point market price for chitosan materials should be 64,400 USD/t, while a lower price could be obtained for chitosan material modified with TiO_2_ nanoparticles (37,000 USD/t) [120]. This economic research has highlighted the sustainability of the chitosan market, which could allow for the establishment of highly profitable businesses if the economic performance parameters for both material topologies are taken into account. The authors concluded that the titanium-dioxide-enhanced chitosan material can withstand several variations in operating costs, suggesting that this configuration may be a reliable choice. In the development of efficient and sustainable adsorbent chitosan gels, it is essential to consider the existence of other organic molecules that may substantially affect the adsorption of BPA. Phenomena such as absorption site competition, chemical interactions, and co-adsorption effects can modify the BPA adsorption [121,122]. Wang et al. investigated the influence of ionic strength on the adsorption of chitosan-based cationic porous polymer towards endocrine disruptors by varying NaCl concentrations from 0 to 10% [115]. The chitosan-based material had the removal efficiency of the four endocrine disruptors chosen, among which BPA, unchanged as the NaCl concentration increased. Also, the authors tested the BPA adsorption efficiency in the presence of humic acid (a frequent organic substance that occurs naturally in water) and can be found in concentrations far higher than those of pollutants and is likely to interfere with the adsorption of endocrine disruptors. The scientists demonstrated that their chitosan-based material exhibited sustained adsorption of BPA with significant anti-interference capacity, indicating its potential use in actual aquatic systems. The competition interactions of BPA with 2,4,6-trichlorophenol during adsorption in chitosan modified with fly ash were investigated in aqueous media [113]. The impact of binary solution systems indicated that BPA adsorption onto modified chitosan materials was reduced via competition for binding sites. The overall adsorption capacity decreased with the rise in competitive 2,4,6-trichlorophenol, but it saw a modest increase in the presence of competitive BPA. Another study tested the efficiency of chitosan-immobilized nanoscale zero-valent iron nanoparticles towards BPA adsorption from pharmaceutical real wastewater [111]. The removal of BPA from pharmaceutical wastewater was 93.8%. This outcome corresponds with the elimination efficiency attained for the synthetic aqueous solution under comparable circumstances, where the removal efficiency for BPA was 95%. The authors attributed the minor difference to the presence of additional contaminants that compete with BPA for adsorption on the active sites of the modified chitosan surface. The presence of additional chemicals in industrial effluents may also influence the adsorption process on the chitosan material surface. The authors examined the efficacy of BPA removal in the presence of coexisting ions, such as SO_4_^2^⁻, NO_3_⁻, Cl⁻, Ca^2^⁺, Mg^2^⁺, Fe^2^⁺, and Mn^2^⁺ with a concentration of 0.1–0.2 mg/mL in the aqueous solutions. The research indicates that inhibitory effects are seen for all examined cations and anions; however, these effects account for a maximum of 5%, allowing the material to retain effective absorption properties for BPA. In conclusion, the experimental procedure validated the nanoparticle-modified chitosan as an efficient agent for industrial wastewater treatment. Tan et al. prepared beads formed by chitosan modified with bone and bamboo biochar and carried out tests for the simultaneous removal of co-existing Cr(VI) and BPA from water [103]. To determine if competitive adsorption occurs when chromium and BPA coexist, competitive adsorption experiments were conducted taking into account the optimal adsorption pH. The authors proved that BPA hardly affects chromium adsorption by competition for active sites. With BPA levels increased, chromium adsorption slightly decreased, an aspect owed to the physical adsorption into chitosan material pores. Similarly, the addition of chromium to BPA had a similar effect on competitive adsorption. In conclusion, the authors underlined that the competitive effect is minor, and no common adsorption mechanism between Cr(VI) and BPA was present except physical adsorption. A molecularly imprinted polymer based on β-cyclodextrin-modified magnetic chitosan was developed for BPA selective removal from waste [98]. The study showed that this material exhibited high recognition capability for BPA, namely, 2,4-dichlorophenol and 3,3,5,5-tetrabromobisphenol A. These compounds have similar functional groups (hydroxyl and benzene ring) and molecular size, but may also have generate steric effects caused by –Cl and –Br, and therefore may influence adsorption. Therefore, it was concluded that the selective recognition mechanism of the novel material may be associated with the interaction between the template and particular functional groups, as well as the compatibility of imprinting cavities with the template molecule in terms of form and size.

The sustainability and economic viability of adsorption-based purification systems hinge upon a crucial process referred to as adsorbent renewal. It involves recycling the adsorbent to gather contaminants once it has reached saturation. Different studies reveal that chitosan-based gels may be used in numerous adsorption/desorption cycles with success. Depending on the absorbent type, for BPA desorption, different solvents may be used, such as pure ethanol, methanol, mixtures of methanol/acetic and diethyl ether/methanol, or NaOH solution. One study showed that methanol was the ideal desorption agent for carbon-fiber-embedded chitosan–PVA composites and that, in reusability tests, the adsorption capacity for BPA drops by around 20% in three cycles and shows no appreciable change in five cycles [27]. Other authors used methanol/acetic acid of BPA from modified chitosan for desorption cycles [113]. They found that the highly competitive adsorption of methanol molecules onto the absorbent surface is producing a BPA adsorption decrease by 15% after the first desorption phase. Moreover, the scientists noted that the total adsorption capacity for BPA dropped in three desorption stages. This might be justified by the potential of the regeneration process producing a decrease in binding site. For chitosan-immobilized nanoscale zero-valent iron nanoparticles, a dramatic drop in efficiency was observed in four cycles [111]. In the first, second, third, and fourth cycles, the adsorption efficiencies were found to be 95, 85, 65, and 40%. Therefore, the authors recommend the adsorbent to be used for BPA removal for up to three cycles. Moreover, successful BPA reuse results were obtained for a material formed from a molecularly imprinted polymer based on magnetic chitosan modified with β-cyclodextrin [98]. After performing six adsorption and desorption cycles with BPA, a slight modification in the absorbent capacity was detected; the adsorption capabilities of modified chitosan toward BPA almost remained at 86% compared with the first time. This finding indicated that the novel adsorbent has the possibility to be recycled circularly in wastewater treatment without considerable loss of adsorption capacity. Other authors used synthetic sewage water for reusability experiments to test the performance of Fe(II)-immobilized chitosan–alginate composite for BPA degradation [97]. The results indicated that BPA removal decreased during the second cycle, reaching a capacity of 60% by the end of the 5th cycle. The efficiency modification was attributed to the iron depletion and structural damage of the polymeric material. One efficient material was proven to be the chitosan-based cationic porous adsorbent that managed to retain 90% of the adsorption capacity for BPA in eight adsorption/desorption cycles [115]. Considering all of the above, the development and use of chitosan-based adsorbent materials presents a strong case for their utilization in the adsorption of bisphenol during wastewater treatment.

## 7. Concluding Remarks and Prospects

This review analyzed the importance of creating and developing effective chitosan-based gel materials for BPA removal from water sources. An extensive examination of the performance and mechanisms of various gel-based materials consisting of chitosan, chitosan and carbon, porous MOFs, natural minerals and polymers, and polysaccharides was conducted, and the effectiveness of BPA removal through absorption was performed. Furthermore, the analysis of kinetic models, isotherms, and influencing variables has provided crucial insights into comprehending the absorption mechanisms of these materials. To support future efforts to mitigate the adverse effects of bisphenol A, we have made some recommendations below:

- Comprehending the prevalence and establishing the quantity and fate of BPA in water bodies and wastewater is essential. The understanding of bisphenol interactions with aqueous matrices containing metals, dissolved organic matter, and cationic and anionic surfactants, etc., may help in shedding light on adsorption/desorption equilibrium using chitosan and chitosan-based gel materials. Nonetheless, more investigations concerning the absorption of BPA from the aqueous phase and the desorption kinetics of BPA from diverse matrices remain insufficient.
- The use of modified gel absorbents based on modified chitosan shows significant promise in BPA removal efficiency. Consequently, research on new functionalized chitosan-based materials tailored specifically for absorption technologies required by wastewater treatment and addressing water quality conditions to improve BPA removal efficacy and minimize costs in practical applications is of great use. The achievement of functionalized chitosan materials for BPA absorption may be carried out via surface modification, pore tuning, and enzyme and active component grafting to introduce a new catalytic function.
- We tried to describe the absorption mechanisms of chitosan-based gels and reviewed the prevalent forces that governed the adsorption of BPA from aqueous media. Most studies regard the adsorption forces qualitatively and discuss their presence through indirect proofs, while the quantitative analysis remains elusive. One way to address this issue is by computational analysis (DFT and MD simulation) that quantitatively describes the molecular interactions and their contributions to the absorption phenomenon. In addition, there is a need for interdisciplinary approaches that integrate surface chemistry, materials science, and physics. Such collaboration should aim to deepen the understanding and provide perspectives on the quantification of the contributions of different forces in the process of adsorption.
- In order to apply the chitosan gel absorbents for bisphenol elimination from water at an industrial scale, forthcoming research must focus on process optimization and technological integration. Therefore, the application of novel materials in current practice requires an assessment of material selection, design complexities, and optimization of operational parameters. Additionally, it is vital to establish control limits, determine the absorbent circularity, and thoroughly dress the entire cost of the industrialization process to ensure the successful practical application of the new absorbent within the established water treatment procedures. To achieve all of the above, industry collaborators could provide expertise for the pilot-scale testing of the new adsorbents in real environments, as well as perform a techno-economic analysis.
- An issue requiring careful consideration is the inadequate adsorption of BPA by chitosan-based gels. Studies concerning this issue are rather scarce. Therefore, it is essential to refine process parameters such as flow rate, temperature, and pH, adjust adsorbent dosages, incorporate supplementary treatment methodologies or technologies, and enhance system design or operational protocols to achieve cleaner water and a healthy environment.
- Finally, significant efforts should be made to address the disposal and regeneration of chitosan adsorbed with BPA given its hazardous characteristics. A non-environmentally friendly way to manage the problem is by creating hazardous landfill waste. An environmentally friendly alternative would be to pursue a sustainable approach that involves the development of recycling and reuse techniques for both BPA and chitosan. Through the development of innovative modifications to chitosan-based absorbents, it is possible to not only enhance absorption but also facilitate the degradation of BPA into non-hazardous and readily extractable compounds from the chitosan gel matrix.

In conclusion, we understand the important effects and possible applications of chitosan-based gels, which offer long-term solutions and follow a green path in bisphenol A removal from water. We hope that this review will help further research aimed at the fabrication and application of chitosan-based adsorbents in the cleaning of polluted water, thus contributing to a cleaner and healthier environment.

## Figures and Tables

**Table 1:** Illustrations of various pollutants subjected to treatment via flocculation using chitosan-derived materials.

Flocculation Agent	Pollutant	Optimal Conditions	Efficiency	Ref.
Chitosan–acrylamide (CS-AM)	Kaolin suspension	pH = 7, CS-AM dosage of 5 mg/L, flocculation time of 30 min, agitation intensity of 250 rad/min and kaolin concentration in the range of 50–100 mg/L	Removal rate can be as high as 95.9%	[58]
Chitosan–polyaluminum ferric silicate	Wool scouring wastewater—chemical oxygen demand (COD)	pH = 5, polyaluminum ferric silicate dosage of 1.2 g/L, chitosan dosage of 10.0 mg/L	Removal rates of turbidity, T-COD and D-COD were 98.7%, 88.8%, and 70.2%, respectively	[59]
Chitosan-based flocculant CS-g-P(AM-IA-AATPAC)	Ni^2+^	pH = 9, flocculant dosage = 4 mg/L, the stirring speed = 400 rpm, and flocculation time = 30 min	Flocculation effect of Ni^2+^ by CS-g-P(AM-IA-AATPAC) was 86.3%	[60]
Chitosan	Vegetable oil refinery wastewater	pH = 6, FeCl3 dosage of 1.6 g/L, chitosan dosage of 13.4 mg/L, and agitation time of 26 min,	Removal rates of 100% turbidity, 86% COD reduction, and 90% polyphenol	[61]
Chitosan-graft-poly(N-vinylcaprolactam) and chitosan-graft-[poly(N-vinylcaprolactam)-poly(acrylic acid)]	AuNP and AgNP	pH = 8, contact time of 60 min, temperature of 45 °C, 0.5 mg/L flocculant dosage.	Nanoparticle removal was above 94%	[62]
Gallic acid-grafted cationic chitosan	Methyl blue (MB) and Congo red (CR)	pH = 5, 3 mg/L of flocculant, and dyes concentration under 60 mg/L	Removal rates of 98.7% for MB and 94.5% for CR	[63]
Chitosan–acrylamide–aluminum chloride (CA-PAC)	Kaolin suspension	pH = 7, CA-PAC dosage was 3 mg/L, and the stirring intensity was 200 rad/min	Removal rate of turbidity was 94.1%	[64]
Chitosan-graft-poly (N, N-Dimethylacrylamide) (CSPD) and chitosan-graft-L-Cyclohexylglycine (CSLC)	Congo red, alizarin green, acid chrome blue k, acid orange 7, and Metanil yellow	pH = 2–9, dosage in the range of 8–70 mg/L	Removal efficiencies by CSPD and CSLC were between 83.17 and 99.40%	[65]
Silver-based metal–organic framework–chitosan (Ag-MOF-CS)	Diclofenac sodium (DCF); acid red 1 (AR1)	-	Removal rate for DCF was 94.8%, while for AR1, it was 94.3%	[66]
Iron-modified chitosan (FeOX-CS)	Oil-water emulsions	pH = 7, contact time of 60 min, FeOX-CS dosage of 20 mg/L, emulsion concentration of 200 mg/L	Emulsification rate excessed 97%	[67]

**Table 2:** Absorption of BPA by chitosan and chitosan-based gels.

Adsorbent and Preparation Type	Removal Capacity at Optimal Condition	Removal Mechanism	Isotherm Models	Kinetic Model	Ref.
Carbon fiber–chitosan–polyvinyl alcohol material obtained by chemically cross-linking with glutaraldehyde	97.6% (0.05 mg/mL BPA adsorbed by 2.8 mg/mL absorbent)	Sorption via H-bond interaction (-OH of adsorbent and BPA molecules) and π–π interactions (π electrons of organic molecules & π electrons of benzene rings or C=C double bonds of carbon material). The adsorption occurs on heterogeneous surface	Pseudo-second-order model, q_e,exp_ = 14.99 mg/g BPA dosage 0–0.05 mg/mL	The Elovich model, α = 137.6 mg/(g min), BPA dosage 0–0.05 mg/mL	[27]
Bentonite–chitosan composite obtained by cross-linking in acidic media	94% (0.02 mg/mL BPA adsorbed by 0.02 mg/mL adsorbent in 60 min)	Chemisorption-based adsorption process	Pseudo-second-order model, q_e,cal_ = 55.38 mg/g, BPA dosage 0.01–0.6 mg/mL	Langmuir isotherm model, Q_max_ = 465.21 mg/g, BPA dosage 0.01–0.6 mg/mL	[96]
Fe(II)–chitosan/alginate obtained by physical cross-linking between polymers	100% (0.01 mg/mL BPA adsorbed by 1 mg/mL adsorbent at pH = 6 in 120 min)	The adsorption and chemical decomposition of BPA takes place concomitantly while the chemisorption may be the rate-determining step	Pseudo-second-order model	-	[97]
β-cyclodextrin modified magnetic chitosan obtained by covalent modification and thin film using surface molecular imprinting	100% (0.02 mg/mL BPA adsorbed by 0.4 mg/mL adsorbent in 60 min at 30 °C and pH = 7)	Adsorption by hydrophobic and electrostatic interactions	Pseudo-second-order model, q_e,cal_ = 63.694 mg/g	Langmuir isotherm model, Q_max_ = 105.5 mg/g, BPA dosage 0–0.6 mg/mL	[98]
Chitosan–bamboo biochar obtained by chemically cross-linking with glutaraldehyde and having a specific surface area of 80.3981 m^2^/g	99.2% (0.005 mg/mL BPA adsorbed by 0.001 mg/mL adsorbent in 180 min at 40 °C)	Chemical adsorption and degradation with H_2_O_2_	First-order kinetics, q_e,eq_ = 58.43 mg/g	Langmuir model, Q_max_ = 140.30 mg/g, BPA dosage 0–1 mg/mL	[103]
Chitosan modified with n-dodecanal mixed with magnetic silica obtained by inverse suspension polymerization and sol-gel method and having a specific surface area of 66.9 m^2^/g	92% (10 mg/mL BPA adsorbed by 5 mg/mL adsorbent in 15 min at pH = 7.0, 25 °C)	Hydrophobic interaction and hydrogen bonding between BPA and composite. multilayer	Pseudo-second-order kinetic model, k_2_ = 0.1838 g/(mg·min)	Freundlich isotherm model, 1/n = 0.8236, BPA dosage 0.01–0.05 mg/mL	[108]
Microspheres with hydrophilic carbonaceous layer on the Fe_3_O_4_ with chitosan coating obtained by cross-linking with EDC·HCl and NHS	97% (0.0005 mg/mL BPA adsorbed by 20 mg/mL adsorbent in 20 min)	-	-	-	[109]
Core–shell chitosan–alginate microcapsules containing tyrosinase obtained by cross-linked with NaTPP of chitosan core with tyrosinase, and cross-linking of alginate shell with glutaraldehyde	84.5% (0.34 mg/mL BPA by 3 adsorbent microcapsules)	Enzymatic degradation of BPA, followed by chemical adsorption of the degraded compound within the core material	-	-	[110]
Chitosan with nanoscale zero-valent iron nanoparticles obtained by with a reduction of ferric iron by NaBH4 in the presence of chitosan as a stabilizer followed by glutaraldehyde cross-linking	93.8%-BPA from real pharmaceutical wastewater (0.006 mg/mL BPA adsorbed by 1.5 mg/mL adsorbent in 60 min at pH = 3)	-	Pseudo-first-order kinetic model, q_e_ = 1.605 mg/g	Langmuir isotherm model, Q_max_ = 65.16 mg/g, BPA dosage 0–0.032 mg/mL	[111]
Magnetic multi-wall carbon nanotubes modified with chitosan obtained by chemical coating	81%-BPA from water (0.0065 mg/mL BPA adsorbed by 0.78 mg/mL absorbent in 91 min at 20 °C)	BPA adsorption process on nanotube biopolymeric surface was spontaneous governed by physical forces	Pseudo-second-order model, q_e,cal_ = 20.58 mg/g	Langmuir isotherm model, Q_max_ = 46.2 mg/g, BPA dosage 0.0065–0.0155 mg/mL	[112]
Chitosan/*γ*-Fe_2_O_3_/fly-ash-cenospheres composites obtained by emulsification process	-	Adsorption process controlled by film diffusion	Pseudo-second-order kinetics, q_e,cal_ = 9.259 mg/g at 35 °C	Langmuir isotherm model, Q_max_ = 103.1 mg/g at 45 °C, BPA dosage 0–0.08 mg/mL	[113]
Supramolecular composite cellulose, chitosan, and cyclodextrin obtained by physical interaction of cellulose, chitosan, and cyclodextrin in an ionic liquid	100% (0.32 mg/mL BPA adsorbed by 0.01 mg/mL adsorbent)	Instantaneous adsorption followed by gradual adsorption where intraparticle diffusion is rate-limiting step	Pseudo-second-order kinetics, q_e_ = 1.59 × 10^−3^ (M/g), BPA dosage 0–1.30 mg/mL	-	[114]
2-hydroxypropyltrimethyl ammonium chloride chitosan grafted with styrene obtained by radical polymerization having a surface area of 806 m^2^ g^− 1^	90% (0.005 mg/mL BPA absorbed by 0.3 mg/mL adsorbent)	Chemisorption-based process. Stage I is the adsorption on the outer surface of the material and stage II is the adsorption on the internal pores. The rate-limiting step of the whole adsorption process determined by film diffusion and intra-particle diffusion	Pseudo-second-order model, q_e,cal_ = 15.6 mg/g	Langmuir model, Q_max_ = 262 mg/g, BPA dosage 0–0.10 mg/mL	[115]

Q_e_ is the adsorbed amounts at equilibrium, Q_e,exp/cal_ is the experimental/calculated value of Q_e_, Q_max_ is the maximum adsorption capacity, α is the initial sorption rate, k_2_ is the rate constant of pseudo-second-order adsorption, 1/n is a constant showing the surface heterogeneity of absorbents.
